# Toward sustainable aesthetic education: exploring the impact of GenAl collaboration on students’ critical thinking disposition

**DOI:** 10.3389/fpsyg.2026.1852109

**Published:** 2026-06-17

**Authors:** Huiying Liu, Tianyue Niu, Chen Shengyan, Patrick Pang, Ting Liu, Yiming Taclis Luo

**Affiliations:** 1 Jimei University, Xiamen, China; 2 Xiamen Academy of Arts and Design, Fuzhou University, Xiamen, China; 3 Faculty of Applied Sciences, Macao Polytechnic University, Macao, Macao SAR, China

**Keywords:** aesthetic education, critical thinking disposition, generative artificial intelligence, randomized controlled trial, thematic analysis

## Abstract

**Introduction:**

Artificial intelligence (AI)-enabled tools have increasingly influenced artistic creation processes in aesthetic education. However, empirical evidence regarding their impact on students’ critical thinking disposition remains limited. This study examined whether integrating Generative Artificial Intelligence (GenAI) tools into an aesthetic learning workshop could enhance undergraduate students’ critical thinking disposition and explored the underlying mechanisms.

**Methods:**

A mixed-methods design was employed. Sixty-three undergraduate arts and design students were randomly assigned to an experimental group (*n = 31*) or a control group (*n = 32*). Both groups participated in a two-week structured landscape sketching workshop, while only the experimental group was permitted to use GenAI tools, including image generation models and large language models, to support idea generation, visual experimentation, and iterative refinement. Critical thinking disposition was measured before and after the intervention using a standardized scale. Quantitative analyses included independent-samples tests and ANCOVA, while qualitative data were analyzed to identify learning mechanisms associated with GenAI use.

**Results:**

The experimental group demonstrated significantly greater improvement in critical thinking disposition than the control group (*p* < 0.01, Cohen’s *d* = 0.70). Dimension-level analyses and ANCOVA further confirmed that these gains remained significant across multiple dimensions of critical thinking after controlling for baseline differences. Qualitative findings revealed three interrelated mechanisms contributing to these improvements: perceptual-conceptual alignment, iterative visual experimentation, and AI-mediated reflective structuring.

**Discussion:**

The findings suggest that GenAI tools can function as cognitive amplifiers in aesthetic learning by promoting more analytical, iterative, and reflective creative practices. This study provides empirical support for the integration of AI tools in design education and offers practical implications for fostering higher-order thinking skills in workshop-based learning environments.

## 1 Introduction

Aesthetic education is a teaching method aimed at cultivating learners’ perceptual sensitivity, evaluative judgment, and reflective engagement with visual and artistic phenomena (Costantino, 2015). Unlike vocational arts training that focuses on developing technical proficiency, aesthetic education emphasizes the development of cognitive and emotional inclinations, enabling individuals to critically interpret, evaluate, and respond to visually relevant information. A lot of scholarship in aesthetic education theory supports the view that artistic practice is fundamentally a form of cognitive inquiry. Eisner argued that artmaking engages qualitative reasoning, which is the capacity to make judgments about relationships, proportions, and expressive qualities that cannot be reduced to fixed rules (Rolling, 2006). Qualitative reasoning requires the perceiver to attend to subtle variations, weigh competing possibilities, and arrive at context. Similarly, Dewey conceptualized artistic creation not as mere self-expression but as a structured form of inquiry in which perception and conception are continuously integrated (Shusterman, 2010).

Critical thinking, as a core higher-order cognitive ability for students, emphasizes the thinking ability that individuals possess when conducting thorough analysis and evaluation of information and forming judgments and decisions (Loyens et al., 2023). Critical thinking disposition, as conceptualized by Facione (2000), refers to the consistent internal motivation to engage in and value critical thinking. It encompasses not only the ability to reason analytically but, more importantly, the habitual inclination to do so across varied contexts. It is not only regarded as a core element for students’ lifelong learning development, but also can significantly promote students’ cognitive development and academic performance, and influence their performance in future practice (Dumitru and Halpern, 2023; Dunlap and Grabinger, 2003; Luo et al., 2026).

In the context of aesthetic education, critical thinking transcends mere logical analysis; it is a necessary ability for individuals to maintain aesthetic autonomy and subjective judgment when faced with complex visual information (Xenakis and Arnellos, 2025). Unlike traditional vocational education, the primary goal of higher aesthetic education is to cultivate students’ spiritual depth and their capacity for emotional resonance. As (Perkins et al., 1993) mentioned in their dispositional theory of thinking, higher-order cognition depends not only on cognitive skills but also on the inclination and sensitivity to deploy those skills in appropriate contexts. Therefore, the essence of aesthetic education lies in placing learners in situations that require these critical tendencies. For example, maintaining an open mind when exploring different visual solutions; demonstrating analytical skills when breaking down complex visual scenes into their constituent elements, etc. (Efland, 2002) further argued that the arts promote cognitive pluralism, which is the ability to operate across multiple representational systems and integrate them in the service of meaning-making. This capacity for cross-domain integration is a hallmark of mature critical thinking, as it requires learners to move beyond single-perspective analysis toward more holistic, multi-faceted evaluation. This provides the theoretical rationale for examining critical thinking disposition as an outcome of aesthetic education.

Aesthetic learning refers to the various learning activities students undertake during artistic practice, including observation, interpretation, creation, and experimentation. It differs from artistic creation in that the former focuses more on the inherent thought processes within the creative process rather than the final material outcome. For this reason, many scholars argue that aesthetic education should help students develop reflective and evaluative thinking habits. In an era where generative artificial intelligence can instantly generate technically “perfect” works of art, the focus of aesthetic education must shift from technical proficiency to the cultivation of critical thinking, enabling students to question and reflect (Jiang et al., 2025). This shift aligns closely with the United Nations Sustainable Development Goal 4 (SDG 4.7), which advocates for education that promotes sustainable development and global citizenship. In the digital society, “sustainability” in aesthetic education refers to students’ cognitive sustainability, ensuring that their creative agency and critical reflection are not undermined by algorithmic reliance (Sclater, 2018).

In traditional aesthetic education, teaching often hits a bottleneck at the stage of art understanding and criticism. However, the emergence of GenAI, such as diffusion models and large language models, has provided art students with a unique conversational partner. GenAI refers to a type of AI technology that can generate brand-new content including text and video by learning from large-scale data (Luo et al., 2025). GenAI enables students to generate dozens of visual prototypes within min and also supports them in conducting quick art experiments on style transfer and fusion. This low-cost experiment encourages students to think divergently (Keser, 2024). From a distributed cognitive perspective (Hutchins, 2020), cognitive processes are not simply confined to individual thinking, but rather distributed among people, objects, and representational media. GenAI tools, by generating visual alternatives, presenting style variations, and providing linguistic feedback on compositional choices, act as external cognitive resources, altering the structure of the creative task itself. Vygotsky and Cole (1978) perspective, GenAI can be seen as a dialogue partner operating within the learner’s zone of proximal development. The iterative prompt-feedback loop between students and GenAI systems reflects the scaffolded instruction process described in sociocultural theory: the output of artificial intelligence provides a reference point that learners need to interpret, critique, and integrate, which precisely requires the qualities that constitute critical thinking.

Despite the great potential of GenAI in aesthetic education, its integration into teaching still faces many challenges. Existing research largely focuses on ethical and governance issues. For example, scholars have expressed concerns about copyright and intellectual property issues related to AI-generated content, and have raised broader ethical questions such as lack of transparency, ambiguity of author identity, and academic integrity (Al-Kfairy et al., 2024; Carobene et al., 2024). In addition, some studies have pointed out that the widespread application of GenAI may inadvertently exacerbate inequalities in access, equity, and cultural representation (Uddin et al., 2025).

However, despite the importance of these ethical discussions, current research still lacks a systematic exploration of the specific teaching practices of GenAI in human-computer interaction (Luo et al., 2025). In other words, most studies focus on whether AI should be used and how its use should be regulated, while paying less attention to how students interact with AI in actual learning processes and how this interaction affects artistic understanding and critical thinking. Therefore, the field of aesthetic education still needs more research centered on the learning process to explore the human-computer collaboration model of GenAI in real teaching situations and its impact on learning experience and cognitive development.

This study enriches the theoretical foundation of collaborative learning using technology in aesthetic education by exploring the relationship between GenAI collaboration and students’ critical thinking tendencies. The findings provide empirical support for aesthetic education curriculum designers and administrators to integrate GenAI into art education. This helps stakeholders create more open and sustainable learning environments in aesthetic education curricula and contributes to the development of higher-order cognitive abilities such as critical thinking among art students. We raised the following questions:

*RQ1*: Does GenAI-supported aesthetic learning significantly enhance undergraduate design students’ critical thinking disposition compared to conventional instruction?

*RQ2*: Through what cognitive mechanisms does GenAI-mediated artistic practice influence students’ critical thinking development?

## 2 Related work

Recent studies show that the rapid development of GenAI is reshaping creative education. Tools such as ChatGPT, Midjourney, and DALL⋅E are increasingly used in visual art, design, music, and interdisciplinary STEAM learning environments (Avlonitou and Papadaki, 2025). Existing literature suggests several major characteristics of GenAI integration in aesthetic education, and we will discuss this in the following subsections.

### 2.1 GenAI as a tool for creative ideation and divergent thinking

A common finding in the literature is that GenAI strongly supports the early stage of artistic ideation, and interaction with generative systems can increase divergent thinking performance, especially in terms of idea fluency and flexibility. Kim shows that generative models can quickly produce visual or conceptual variations, which helps students explore different creative directions. Students often use these outputs as inspiration during brainstorming and concept development stages (Avlonitou and Papadaki, 2025). Lee reveals that students generate more design alternatives when they use GenAI tools during the early phase of creative tasks (Lee, 2025). Especially, this effect appears particularly beneficial for novice learners. Beginners often face difficulties when they try to start a creative project. GenAI can reduce this barrier by offering initial ideas and visual references that help students enter the creative process more easily (Chu et al., 2025).

### 2.2 The ideation–execution gap in GenAI-assisted artistic creation

Another problem in the GenAI-assisted artistic creation is the gap between idea generation and artistic execution. GenAI performs well in generating conceptual ideas and stylistic variations. However, many studies report that students still encounter limitations when they try to translate GenAI outputs into refined artworks, including insufficient control over composition, color consistency, or spatial details (Jang, 2026). The effectiveness of GenAI tools tends to decrease when tasks require fine artistic judgment or technical execution. As a result, students often rely on AI during brainstorming but return to manual work during the final production stage (Jang, 2026). At this stage, most researchers argue that GenAI should therefore be viewed as a collaborative assistant rather than a replacement for human creativity. Human interpretation and artistic decision-making remain necessary during the execution stage (Wu et al., 2026).

### 2.3 Cognitive and affective impacts on artistic learning

Beyond technical support, several studies report psychological and learning-related effects of GenAI use. Quantitative studies suggest that GenAI-supported learning environments may enhance some dimensions of creativity. For example, experimental research using creativity assessment tools reports higher scores in fluency and flexibility among students who interact with GenAI systems (Fan and Zhong, 2022; Xu, 2025). GenAI may also influence students’ emotional experiences during artistic learning. Some studies report that conversational AI systems can reduce performance anxiety and create a more relaxed learning environment (Mirriahi et al., 2025). Students also report higher levels of learning motivation and self-efficacy when they receive immediate feedback or suggestions from AI systems. These effects appear particularly strong in exploratory or studio-based learning contexts (Fang, 2026).

While these studies have highlighted the cognitive and affective benefits of GenAI in art learning, several important research gaps remain. First, existing research primarily focuses on outcomes related to creativity, with relatively few practical measurements of higher-order cognitive abilities such as critical thinking tendencies. The vast majority of existing research relies on quantitative measurements or self-reported perceptions (Bailey et al., 2026), while studies employing mixed approaches to simultaneously examine the mechanisms at both the outcome and process levels are scarce. Second, although previous research has shown that GenAI can influence learners’ emotional and motivational states, the underlying processes by which these influences translate into students’ cognitive development remain insufficiently explored. In particular, our understanding of how GenAI-supported interactions shape students’ analytical, iterative, and reflective thinking during the creative process remains limited.

To fill these research gaps, this study employs a combination of randomized controlled experimental design and thematic analysis to explore the impact of GenAI-supported aesthetic education on students’ critical thinking tendencies. By integrating quantitative and qualitative evidence, this study aims to provide a more comprehensive understanding of how GenAI assists in the outcomes and processes of art learning.

## 3 Materials and methods

### 3.1 Participants

Sixty-three undergraduate students majoring in design and fine arts were recruited from Jimei University in Fujian Province. Participants were recruited from two departments within the School of Art at Jimei University through social media platforms. Participants were randomly assigned to two groups by lottery, with a ratio of 31:32 between the experimental and control groups. Baseline data were collected from both groups before the intervention to test the effectiveness of randomization. Independent samples *t*-tests and chi-square tests were conducted on several key variables, including design foundation course grades, gender, and years of art study. Results (Table 1) showed no significant difference between the two groups (*p* > 0.05). This indicates that randomization was effective and the two groups were comparable.

**TABLE 1:** Demographic table of participants.

Variable	Control group (*n* = 32)	Experimental group (*n* = 31)	Statistical test	*p*-value
Age (years)				
Mean ± SD	20.65 ± 1.28	20.88 ± 1.15	*t* = -0.75	0.456
Gender, n (%)				
Male	14 (43.8%)	14 (45.2%)	χ^2^ = 0.01	0.911
Female	18 (56.2%)	17 (54.8%)		
Years of design study, n (%)				
≤1 year	11 (34.4%)	11 (35.5%)	χ^2^ = 0.01	0.926
>1 year	21 (65.6%)	20 (64.5%)		
GPA in foundation courses				
Mean ± SD	3.51 ± 0.26	3.49 ± 0.25	*t* = 0.31	0.758

*p < 0.05, **p < 0.01, ***p < 0.001.

After the intervention, 31 students in the experimental group and 32 students in the control group completed the entire research process. This study adhered to academic ethical guidelines. The ethics committee of the host university approved the study before its commencement. All participants signed informed consent forms, clearly explaining the research objectives, procedures, and the right to withdraw from the study at any time. All data were anonymized and kept strictly confidential throughout the research process.

### 3.2 Experimental task

An observational landscape sketch was assigned as the experimental task. Unlike purely technical drawing exercises (such as figure drawing), observational landscape sketching requires learners to engage in multiple cognitive processes directly related to critical thinking (Swailes, 2010). These processes include: perceptual analysis, which means breaking down a complex, unstructured visual scene into elements with compositional significance; interpretive judgment, which means deciding what to emphasize, simplify, or omit based on expressive intent; compositional reasoning, which means organizing selected elements into a coherent spatial and tonal structure; and reflective evaluation, which means assessing whether the final work achieved the intended aesthetic and communicative effect. These processes are closely related to what (Rolling, 2006) calls the qualitative intelligence specific to art tasks.

Furthermore, observational landscape sketching is also suitable for testing the cognitive effects of GenAI integration because it involves the transformation between perception (what students observe) and representation (what students create), creating space for thinking and decision-making (Brice, 2024). By offering suggestions for compositional changes or guiding students to articulate their expressive choices, the GenAI tool provides an opportunity to transform this gap between perception and expression from a source of uncertainty into structured reflection and critical evaluation.

### 3.3 Experimental procedure

**FIGURE 1:**
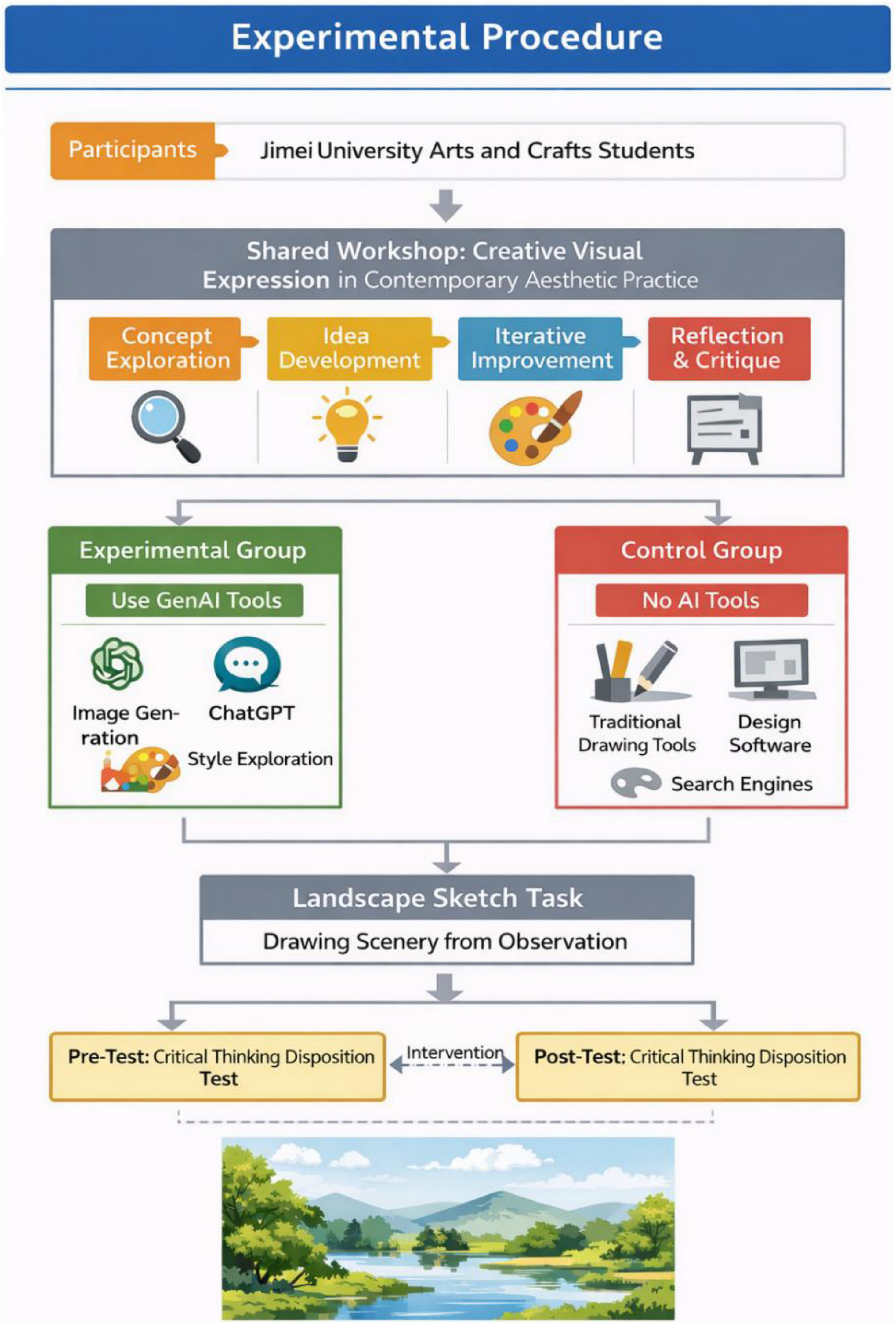
Design process of the experiment.

This study used a pre-test and post-test experimental design. The aim was to examine changes in students’ critical thinking disposition during aesthetic learning. Figure 1 illustrates the relevant process of the entire experimental design: The participants were students who studied arts and crafts at Jimei University. Both the experimental group and the control group joined a 2-week workshop titled “Creative Visual Expression in Contemporary Aesthetic Practice- landscape sketch”. The workshop guided students through a structured process of artistic exploration. The process included four stages: concept exploration and visual inspiration, idea development and visual composition, iterative improvement of artistic concepts, and reflection and critique of the final artwork. The first and second authors served as instructors for the workshop intervention, sharing responsibility for teaching and organization with a research teaching assistant. The remaining authors were responsible for pre- and post-test management, interview data collection, and analysis.

**FIGURE 2:**
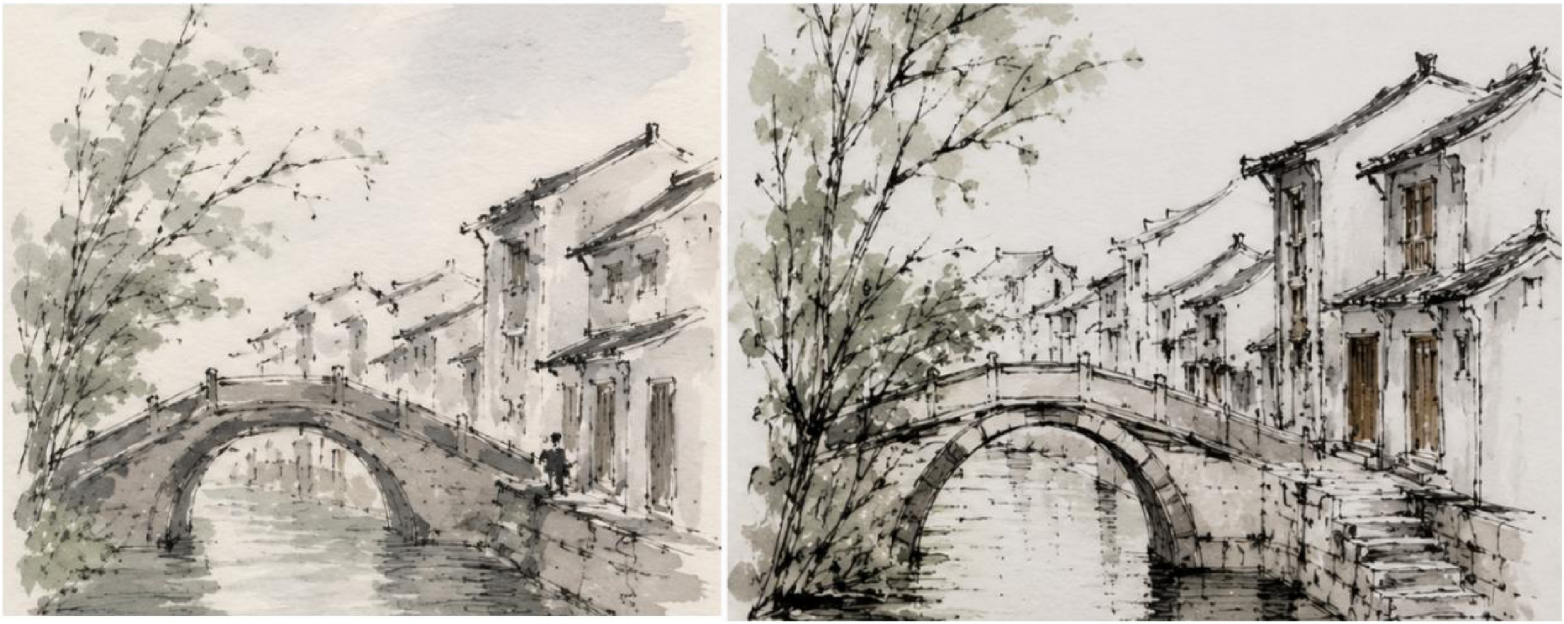
Examples of student landscape sketches.

After the workshop intervention, the researchers assigned a landscape sketch task. The theme of the task was drawing scenery from observation. The key difference between the two groups was whether students could use GenAI tools during the creative process. Students in the experimental group were allowed and encouraged to use GenAI-based tools. These tools included image generation models and large language models such as ChatGPT. Students used these tools to support their artistic exploration. The tools helped with idea generation, visual prototyping, style exploration, and iterative improvement of artworks. Students in the control group joined the same workshop. The theme, duration, and teaching structure were the same. However, these students were not allowed to use any AI-based tools. They could only use traditional digital drawing tools, search engines, or design software to complete their tasks. Before the workshop began, all participants completed a pre-test. The pre-test measured their baseline level of critical thinking disposition. After the intervention ended, the researchers collected the students’ work (see Figure 2) and conducted a post-test assessment using the same scale again.

### 3.4 Quantitative analysis

The researchers first used paired statistical tests to check whether the pre-test and post-test scores changed within each group.

When the data met the normal distribution assumption, the researchers used a paired-sample *t*-test. When the data did not meet this assumption, the researchers used the Wilcoxon signed-rank test. The researchers also wanted to measure the overall effect of the GenAI-supported intervention. They therefore used analysis of variance (ANOVA). This analysis compared the post-test scores of the experimental group and the control group, while controlling for the pre-test scores. In addition, the researchers calculated the difference between pre-test and post-test scores. This value showed how much students’ critical thinking disposition improved.

### 3.5 Qualitative data collection and analysis

This study also collected qualitative data through semi-structured interviews. The goal was to better understand students’ experiences when they used GenAI tools in artistic learning. All students in the experimental group joined an interview that lasted about 10–15 min after the workshop ended to minimize potential influence on scale responses. The interview focused on their human-AI collaborative creation experience. The questions asked about several topics: (1) how GenAI influenced their idea generation, the role of GenAI in visual experiments and aesthetic exploration, (2) how GenAI affected their critical reflection during the creative process, and (3) the challenges they faced when working with AI tools.

**FIGURE 3:**
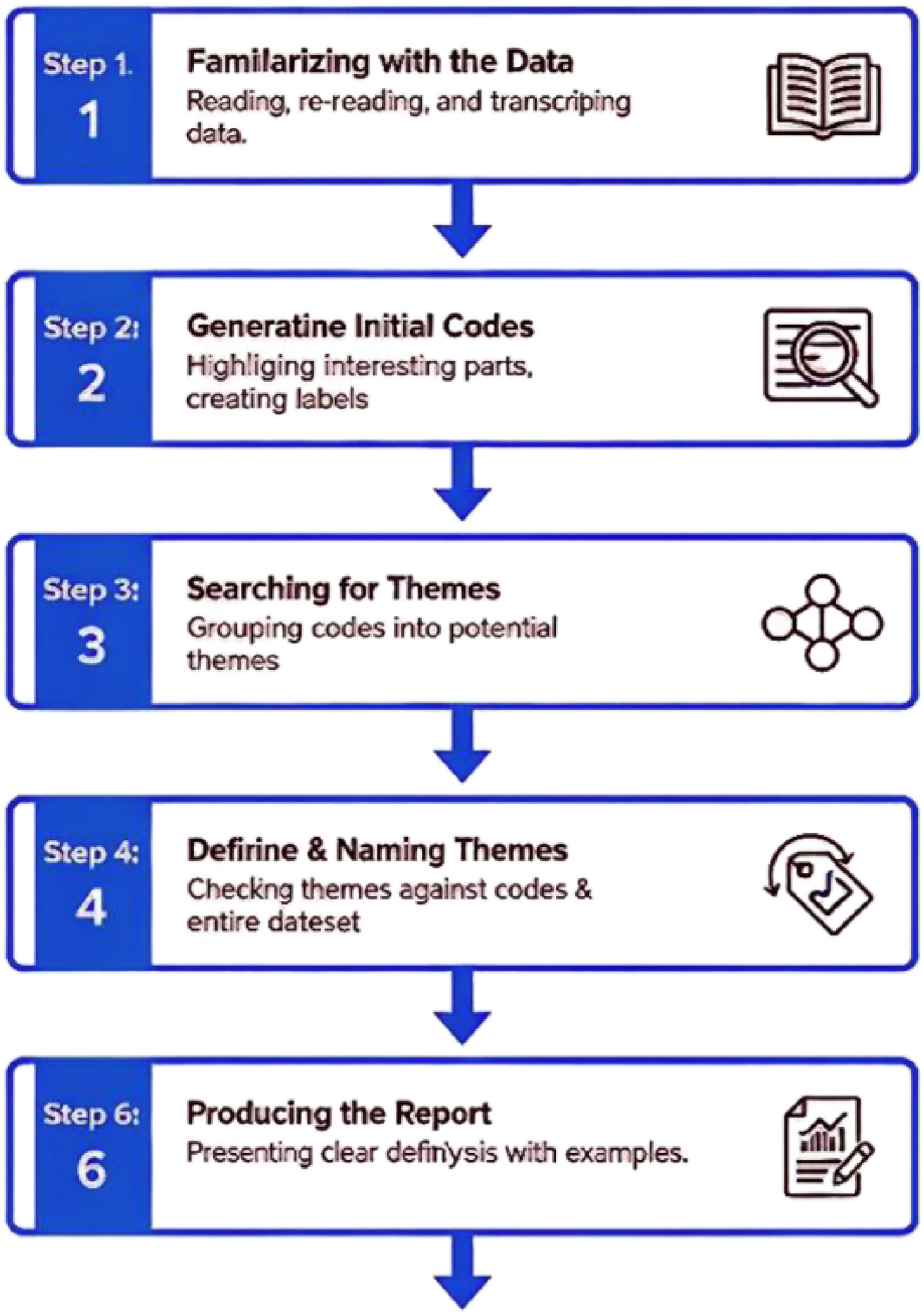
Six-step framework.

The interview used open-ended questions. This design allowed participants to describe their ideas in detail. The researchers analyzed all interview transcripts with MAXQDA software. They used thematic analysis to examine the data. The analysis followed the six-step framework, which is shown in Figure 3 proposed by (Braun and Clarke, 2006).

In the first phase, two researchers (two of the authors) repeatedly reviewed the interview transcripts and familiarized themselves with the data to gain a comprehensive understanding of the participants’ responses to the semi-structured interviews. In the second phase, the researchers inductively generated initial codes. They identified and labeled text fragments related to students’ critical thinking tendencies (e.g., assessment, reflection, and decision-making). The coding process was primarily data-driven but also referenced theoretical dimensions of the critical thinking tendencies scale. In the third phase, the researchers organized the codes into potential themes by identifying patterns and relationships among the participants. Similar codes were grouped together to form candidate themes that could capture recurring critical thinking tendencies.

In the fourth phase, the researchers reviewed and refined these themes by examining their internal consistency and differentiation from one another, ensuring that each theme was strongly supported by multiple data extracts. To extract as many compact concepts as possible, the researchers refined these concepts as much as possible during the organization phase. In the fifth phase, the themes were clearly defined and named. Each theme was conceptualized to reflect the specific mechanisms by which GenAI influences students’ critical thinking tendencies. Finally, in the sixth phase, these themes were integrated into a coherent analytical narrative, supplemented by representative quotations from the participants.

To ensure the reliability and effectiveness of the coding process, we employed several strategies. First, we conducted dual coding. Two researchers independently coded portions of the interview transcripts (approximately 20% of the total duration) and assessed the consistency between the coders. Disagreements were discussed and resolved through consensus, thus refining the coding scheme. Subsequently, we applied the revised coding framework to the complete dataset, and a third author reviewed the coding structure and topic interpretations again to ensure its credibility and reduce potential bias.

This qualitative analysis helped the researchers identify common themes about critical thinking development in GenAI-supported aesthetic creation. The qualitative results also supported and explained the findings from the quantitative analysis.

### 3.6 Instruments

In this study, the Chinese version of the California Critical Thinking Disposition Inventory (CTDI-CV) was employed as the primary instrument to assess changes in the critical thinking dispositions of Chinese students before and after the intervention (Huang et al., 2021). We have created a version specifically designed for Chinese art and design students (Yang and Niu, 2026). The original English CTDI-CV scale is a standardized 70-item multiple choice test, which comprises seven dimensions: truth-seeking, open-mindedness, analyticity, systematicity, critical thinking, self-confidence, inquisitiveness, and cognitive maturity.

To ensure the instrument’s relevance to the theme of GenAI-collaborative aesthetic education, the dimensions were refined and optimized through expert reviews and focus group discussions. Consequently, five dimensions most closely aligned with design research were retained: Open-mindedness, Analytical Thinking, Systematic Thinking, Confidence, and Cognitive Maturity. “Truth-seeking” and “Inquisitiveness” were excluded as they did not directly align with the collaborative nature of the tasks.

To better adapt the tool to the cultural and academic context of the design discipline while streamlining the process, we utilized a 5-point Likert scale, ranging from 1 “Strongly Disagree” to 5 “Strongly Agree,” and three representative items were selected for each dimension (totaling 15 items), with minor linguistic adjustments made to the phrasing. Recognizing that reducing the number of items might impact on the instrument’s psychometric properties, a pilot study was conducted to re-evaluate its validity and reliability. The Scale-level Content Validity Index (S-CVI) was 0.86, indicating that the scale possesses strong representativeness and professional applicability. Reliability analysis revealed a global Cronbach’s α coefficient of 0.88. While the α coefficients for the individual subscales ranged from 0.64 to 0.78, slightly lower due to the limited number of items per dimension (3 items each), they remained within the acceptable range for exploratory research (satisfying the standard of > 0.6) (Adamson and Prion, 2013). This demonstrates that the modified instrument maintains acceptable internal consistency.

## 4 Results

### 4.1 Randomized controlled trial

This section reports on the statistical analysis of students’ critical thinking disposition scores. Table 2 presents the descriptive statistics for both groups across pre-test and post-test. The results indicate that the experimental group demonstrated a notable increase in mean scores from pre-test (*M* = 52.42, SD = 6.72) to post-test (*M* = 56.19, SD = 6.25), with an average gain of 3.77 points. In contrast, the control group showed only a marginal improvement, increasing from 50.62 (SD = 6.39) to 51.53 (SD = 7.04), with a mean difference of 0.91 points. Beyond the difference in mean scores, the score distribution pattern also provides more information. The experimental group had a wider range of scores (0–16), suggesting that the intervention may not have produced a uniform effect but rather amplified individual differences, potentially benefiting some students more than others, requiring further statistical testing.

**TABLE 2:** Descriptive statistics for pre-test and post-test scores.

Group	*n*	Pre-test Mean ± SD	Post-test Mean ± SD	Mean difference	Range of difference
Control	32	50.62 (6.39)	51.53 (7.04)	0.91	[-7, 6]
Experimental	31	52.42 (6.72)	56.19 (6.25)	3.77	[0, 16]

As shown in Table 3, we further performed Within-Group Comparisons on the front and back side results. Given that the normality assumption for difference scores was violated (Shapiro–Wilk test, *p* < 0.05), Wilcoxon signed-rank tests were conducted. For the control group, although a slight increase was observed, the difference between pre-test and post-test scores did not reach statistical significance (*p* = 0.0698). This suggests that, in the absence of GenAI support, the observed improvement may primarily reflect natural variation or a mild practice effect, rather than a systematic developmental change of critical thinking. In contrast, the experimental group showed a statistically significant improvement (*p* < 0.001), indicating that students’ critical thinking disposition increased following the intervention. However, it is important to interpret this finding cautiously. A significant within-group change does not, in itself, establish the relative effectiveness of the intervention, as such changes may still be influenced by factors including regression to the mean or differential engagement levels.

**TABLE 3:** Paired comparisons between pre-test and post-test scores.

Group	Normality test (Shapiro–Wilk)	Statistical test	*W*	*p*-value	Conclusion
Control	*W* = 0.879, *p* = 0.002	Wilcoxon signed-rank test	0.57	0.07	Not significant
Experimental	*W* = 0.855, *p* = 0.001	Wilcoxon signed-rank test	0.0	< 0.001	Significant (Post-test > Pre-test)

To address this limitation, an independent samples *t*-test was conducted on post-test scores. The results revealed that the experimental group scored significantly higher than the control group, with a moderate-to-large effect size (*p* = 0.007, Cohen’s *d* = 0.70, as shown in Table 4).

**TABLE 4:** Independent samples *t*-test comparing post-test scores between groups.

Group	Mean (SD	*t*	*p*-value	Cohen’s *d*
Control	51.53 (7.04) 56.19 (6.25)	2.78	0.007	0.70
Experimental				

Levene’s test indicated that the assumption of homogeneity of variance was met, *W* = 0.43, *p* = 0.514. Therefore, the standard independent samples *t*-test was used. The results show that the experimental group scored significantly higher than the control group on the post-test.

This finding provides stronger evidence that the observed improvement in the experimental group is unlikely to be solely attributable to time-related effects. On the contrary, it shows that the integration of GenAI collaboration into learning brings an additional advantage in critical thinking tendencies compared to the control group’s non-GenAI collaborative learning approach.

To further strengthen causal inference, an analysis of covariance (ANCOVA) was conducted, controlling for pre-test scores. The result is shown in Table 5. It showed that the pre-test score was a strong predictor of post-test performance (*B* = 3.07, SE = 0.83, *p* < 0.001), confirming that baseline ability plays a substantial role in shaping learning outcomes. More importantly, after adjusting for these baseline differences, the group effect remained statistically significant. The effect size (partial η^2^ = 0.184) indicates a substantial practical impact, suggesting that the intervention accounts for a meaningful proportion of variance in post-test scores.

**TABLE 5:** ANCOVA regression results.

Predictor	*B*	SE	*t*	*p*-value	95% CI
Intercept	6.62	3.29	2.01	0.049	[0.04, 13.21]
Group (experimental vs. control)	3.07	0.83	3.68	< 0.001	[1.40, 4.74]
Pre-test score	0.89	0.06	13.86	< 0.001	[0.76, 1.02]

The five dimensions of critical thinking were further analyzed. Table 6 showed a consistent pattern: significant improvements were observed across all five dimensions in the experimental group (*p* < 0.01). The control group showed no significant change in any dimension. This pattern has theoretical implications. The intervention does not appear to affect a single cognitive component, but rather has a broad impact on multiple levels of critical thinking, suggesting that GenAI tools may serve as a multi-layered cognitive scaffold, supporting interpretation, reasoning, and reflective judgment simultaneously.

**TABLE 6:** Dimension-level pre–post comparisons by group.

Dimension	Group	Pre-test mean	Post-test mean	*p*-value	Significant
Open-mindedness	Control	3.31	3.41	0.143	No
	Experimental	3.55	3.76	0.005	Yes
Analytical thinking	Control	3.41	3.45	0.550	No
	Experimental	3.60	3.90	0.005	Yes
Systematic thinking	Control	2.71	2.79	0.122	No
	Experimental	2.99	3.30	0.005	Yes
Confidence	Control	3.69	3.74	0.106	No
	Experimental	3.75	3.92	0.007	Yes
Cognitive maturity	Control	3.76	3.79	0.678	No
	Experimental	3.58	3.84	0.005	Yes

*p*-values are based on paired comparisons within each group.

The experimental group showed significant improvement in all five dimensions (*p* < 0.01). The control group showed no significant change in any dimension. This pattern has theoretical implications. The intervention does not appear to affect a single cognitive component, but rather has a broad impact on multiple levels of critical thinking, suggesting that GenAI tools may serve as a multi-layered cognitive scaffold, supporting interpretation, reasoning, and reflective judgment simultaneously.

### 4.2 Qualitative analysis results

To further interpret the quantitative findings, a thematic analysis was conducted to examine how GenAI-supported artistic practices influenced students’ critical thinking development. Three interrelated themes emerged from the data: (1) Perceptual–Conceptual Alignment, (2) Iterative Visual Experimentation, and (3) AI-Mediated Reflective Structuring.

#### 4.2.1 Perceptual–conceptual alignment

The first theme highlights how GenAI tools facilitated the alignment between students’ visual perception and conceptual understanding. In traditional observational drawing, students often rely on intuitive perception without fully articulating the underlying structure of what they see. However, interaction with GenAI prompted students to reinterpret visual scenes in a more structured and analytical manner. As shown in Table 7, several participants reported that GenAI-assisted prompts helped them notice previously overlooked elements such as spatial relationships, temporal interaction, and functional details:

**TABLE 7:** Student reflections illustrating perceptual-conceptual alignment.

Student	Statement	Interpretation
S2	“I initially thought that making the sky melancholic only required working with light and color, but I overlooked the fact that elements like white clouds could express my emotions more naturally.”	This reflection indicates a shift from intuitive visual judgment may toward more structured perceptual analysis, suggesting developing analyticity.
S9	“I didn’t see the little figures when I was drawing, but after I finished, I realized that the little figures seemed to bring the painting to life.”	Recognizing the importance of previously overlooked spatial elements

#### 4.2.2 Iterative visual experimentation

The second theme reflects how GenAI enabled a process of continuous visual exploration and experimentation. Several students described engaging in repeated cycles of idea generation, testing, and refinement. As shown in Table 8, this iterative process supported the development of analyticity, open-mindedness, systematicity, and critical thinking self-confidence.

**TABLE 8:** Student reflections illustrating iterative visual experimentation.

Student	Statement	Interpretation
S6	“I kept making different versions and looking at them side by side. The one I liked at first wasn’t always the best once I saw the others.”	Actively comparing alternatives reflects analyticity and increased open-mindedness toward revising initial judgments.
S14	“I didn’t directly adopt the initial idea. I modified it little by little, adjusting some details each time, until it felt right.”	Stepwise, deliberate refinement indicates the development of systematicity in the creative process.
S15	“After trying several different versions, I really gained confidence in my final choice (laughs).”	Grounding decisions in generated evidence rather than intuition reflects growing critical thinking self-confidence.

#### 4.2.3 AI-Mediated reflective structuring

The third theme captures how GenAI functioned as a mediating tool that supported students’ reflective thinking and the structuring of complex ideas, which was closely related to multiple dimensions of critical thinking disposition. Students reported that AI-assisted interactions helped them organize fragmented ideas, clarify relationships between visual elements, and articulate their reasoning more explicitly during the creative process. As shown in Table 9, this process was closely associated with multiple dimensions of critical thinking disposition, particularly analyticity, systematicity, and open-mindedness:

**TABLE 9:** Student reflections illustrating AI-mediated reflective structuring.

Student	Statement	Interpretation
S8	“When I tried to explain my thoughts to the AI, I felt some parts were off-key, it always felt like I wasn’t answering the question. So I thought about how to get the clumsy GenAI to understand what I was saying.”	Recognizing internal inconsistencies and reorganizing elements reflects both analyticity and systematicity.
S17	“It kept asking me why I put certain things in the picture, so I had to actually think about my reasons.”	Being prompted to justify design choices led students to examine their own assumptions, reflecting greater open-mindedness and deeper reflective engagement.

This indicates that students became more actively engaged in questioning their own assumptions and examining the reasoning behind their design choices, as students engaged more actively in questioning their own assumptions. At the same time, the process of justifying design choices reflects greater open-mindedness, as students became more willing to reconsider and revise their initial ideas.

## 5 Discussion

### 5.1 Effects of GenAI on critical thinking disposition

The present study provides evidence that GenAI-supported aesthetic learning may positively influence students’ critical thinking disposition. We discovered three insights from the quantitative analysis:

(1) First, improvement is not automatic. The control group’s non-significant change indicates that participation in the workshop alone does not guarantee measurable gains in critical thinking disposition. This finding aligns with prior research indicating that creative practice, when not explicitly scaffolded, may remain at an intuitive or experience-based level rather than developing into higher-order cognitive engagement (Yang and Niu, 2026).

Second, GenAI produced a significant instructional effect. The experimental group demonstrated greater improvement than the control group after controlling for baseline differences, suggesting that GenAI contributes cognitive value beyond conventional instruction. Rather than functioning solely as a productivity-enhancing tool, GenAI appears to support more reflective and evaluative engagement during artistic creation (Han and Lim, 2025).

Third, the intervention introduces variability rather than uniform gains. The wider dispersion in the experimental group suggests that GenAI may act as an amplifier of individual learning trajectories, potentially benefiting students who are more capable of leveraging feedback, iteration, and exploration. Several moderating factors may explain this variability. Cognitive capacity and metacognitive awareness, which is students’ ability to monitor and regulate their own thinking, likely influence the extent to which they can transform AI-generated alternatives into structured reflection. Additionally, prior domain knowledge in visual arts and familiarity with generative tools may shape how effectively students leverage the affordances of GenAI. Individual differences in openness to feedback and tolerance for iterative refinement could determine whether variability manifests as productive exploration or surface engagement. The moderate to large effect size observed (Cohen’s *d* = 0.70) further highlights the practical significance of the intervention in the context of arts education.

Thematic analysis of student experiences revealed how GenAI fosters critical thinking. The study identified three interrelated mechanisms that promote the development of critical thinking tendencies:

(1) GenAI tools helped students link observed visual elements with conceptual understanding. Students reported that AI-generated prompts encouraged them to reinterpret and refine visual observations, facilitating higher levels of analyticity and open-mindedness. This mechanism reflects the foundational step of translating perceptual experience into structured knowledge. This mechanism aligns with distributed cognition theory, in which AI-generated cues, acting as external representation artifacts, reconstruct the observation task, transforming it from passive perception into active interpretation (Pietarinen and Shi, 2026).
(2) GenAI facilitated iterative experimentation and evaluation. Through rapid prototyping and style exploration, students repeatedly compared alternatives, revised decisions, and reflected on design quality. The visibility of multiple design options, which becomes concrete through AI generation, externalizes some of the deliberation processes that are usually invisible, enabling novices to internalize evaluation criteria through concrete comparisons and feedback loops (Yu, 2025).
(3) In artistic education context, GenAI acted as a reflective scaffold, prompting students to organize their ideas, justify design choices, and critically assess the internal logic of their compositions. This scaffolding function echoes some theories of the zone of proximal development: students can use it to express implicit aesthetic judgments and gradually shift from externally supported reflection to internal critical evaluation (Adejumo et al., 2026).

Taken together, these results suggest that GenAI’s role in this context may extend beyond improving the efficiency of artistic creation, potentially serving as a cognitive scaffold that supports analytical, iterative, and reflective thinking by restructuring the art learning process.

### 5.2 Implications

The findings provide several practical and theoretical implications. From an instructional perspective, the integration of GenAI into art education should emphasize structured exploration, guided reflection, and iterative refinement rather than efficiency-oriented content generation alone. Educators can design activities that encourage students to question initial interpretations, compare alternative visual solutions, and justify artistic decisions during the creative process.

The findings also suggest that GenAI-assisted learning may produce uneven outcomes across students. Therefore, personalized guidance and formative feedback remain important to help learners effectively engage with AI-supported reflection and exploration. Theoretically, this study contributes to understanding GenAI-supported learning at the process level. The findings indicate that generative tools may transform traditionally intuitive artistic practices into more reflective and evaluative learning experiences characterized by continuous iteration and critical examination.

### 5.3 Limitation

Although the research results are encouraging, several limitations of this study still need to be pointed out. First of all, the samples are only from the student group majoring in art and design. This limits the general applicability of the research results to other related disciplines, age groups or cultural backgrounds. Secondly, this study focused on short-term intervention (a 2-week workshop) and measured critical thinking tendencies immediately after the intervention ended. The maintenance of long-term effects and whether they can be transferred to other artistic or non-artistic tasks remain to be studied.

Although this study used ANCOVA to control the pretest scores, the differences among individuals in terms of previous experience, motivation, and technical proficiency might have affected the extent of the benefits brought by GenAI. The differences in the results of the experimental group indicate that some students may benefit more than others, depending on the above factors. Fourth, qualitative data is derived from some participants and relies on self-reported reflections, which may be influenced by social expectation bias or recall bias.

Also, it is important to acknowledge the potential influence of the novelty effect. Because the experimental group was aware that they were using AI tools during the workshop, their heightened awareness of the intervention itself may have contributed to increased motivation or engagement, thereby inflating post-survey scores independently of the cognitive scaffolding provided by GenAI. This potential bias cannot be fully ruled out within the current design, and future studies employing blind or active-control conditions would help to disentangle novelty-driven effects from genuine learning gains. The impact of GenAI on critical thinking tendencies may also vary depending on the cultural context that emphasizes different learning values.

Moreover, qualitative analysis is susceptible to interpretive bias. Students’ reflections may emphasize certain cognitive processes while neglecting others, or express hindsight rationalization rather than genuine critical insight. Future research that employs process tracking methods and reflective behavior indicators for longitudinal evaluation after intervention will help to more effectively demonstrate the depth and persistence of critical thinking development.

Observational or performance-based measurement methods can complement self-reporting, thereby providing a deeper understanding of the AI-assisted learning process. Finally, this study examined specific GenAI tools and tasks. Different types of creative tasks or AI interfaces may produce different results, which limits the promotion of research findings to other GenAI-supported learning scenarios. Future research should address these limitations by incorporating larger and more diverse samples, extending the intervention period, conducting longitudinal tracking, and exploring the interaction between learner characteristics and GenAI-assisted learning strategies.

## 6 Conclusion

This study investigated the effects of GenAI-supported aesthetic learning on design students’ critical thinking disposition. The findings indicate that GenAI enhances students’ overall critical thinking scores. Quantitative analyses revealed significant gains in the experimental group compared to the control group, while qualitative insights highlighted the mechanisms through which GenAI supports learning: aligning perception with conceptual understanding, enabling iterative visual experimentation, and scaffolding reflective structuring of ideas. The results suggest that, within this specific context, GenAI may function as a cognitive amplifier, supporting a shift in artistic practice toward more analytical, iterative, and reflective engagement. At the same time, the observed variability in learning gains underscores the role of individual differences in shaping the impact of AI-supported interventions. In conclusion, our research provides preliminary empirical support for cultivating higher-order thinking skills through GenAI-assisted aesthetic learning, offers relevant suggestions for stakeholders to conduct further instructional development, and contributes to the development of the discipline.

## Data Availability

The raw data supporting the conclusions of this article will be made available by the authors, without undue reservation.
